# Blood pressure profile and endothelial function in restless legs syndrome

**DOI:** 10.1038/s41598-019-52401-4

**Published:** 2019-11-04

**Authors:** Sofiene Chenini, Anna Laura Rassu, Lily Guiraud, Elisa Evangelista, Lucie Barateau, Regis Lopez, Isabelle Jaussent, Yves Dauvilliers

**Affiliations:** 1 Sleep-Wake Disorders Center, Department of Neurology, Gui-de-Chauliac Hospital, University Hospital center, Montpellier, France; 2 National Reference Network for Narcolepsy, Gui-de-Chauliac Hospital, University Hospital Center, Montpellier, France; 3 INSERM 1061, University of Montpellier, Neuropsychiatry: Epidemiological and Clinical Research, Montpellier, France

**Keywords:** Movement disorders, Sleep disorders

## Abstract

Restless legs syndrome (RLS) is frequently comorbid with hypertension and cardiovascular diseases; however this relationship and underlying mechanisms remain controversial. After clinical evaluation, 84 drug-free patients with primary RLS (53 women; mean age 55.1 ± 12.3 years) and 76 controls (47 women; mean age 52.2 ± 15.3 years) underwent 24-hour ambulatory blood pressure (BP) and polysomnographic monitoring, and peripheral arterial tonometry to assess endothelial function for 61 patients and 69 controls. Hypertension was diagnosed in 11.9% of patients with RLS based on office measurement, and in 46.4% on the 24 h recording, with nighttime hypertension, two times more frequent than daytime hypertension. Periodic limb movement during sleep (PLMS), markers of sleep fragmentation, and systolic and mean BP non-dipping profile were more frequent among patients. BP non-dipping status was associated with older age, later RLS onset and diagnosis, RLS severity and higher sleep fragmentation. The mean 24-hour, daytime and nighttime BP values, the frequency of hypertension and the endothelial function were comparable between groups. However, both systolic and diastolic BP trajectories over a 24-hour period differed between groups. In conclusion, patients with RLS exhibit a 24-hour BP deregulation with increased frequency of systolic non-dipping profiles that could worsen the risk for CVD morbidity and mortality.

## Introduction

Restless legs syndrome (RLS), also known as Willis-Ekbom disease, is a common neurological sensorimotor disorder defined by uncomfortable sensations in the legs, that worsen in the evening and at night and may result in sleep disturbances^1,2^. RLS is frequently comorbid with obesity, hypercholesterolemia, diabetes mellitus, obstructive sleep apnea, hypertension and cardiovascular diseases (CVD)^3–5^. Recent large epidemiological studies reported an independent associations between RLS and CVD or hypertension; however, this relationship remains controversial^6–10^.

Periodic limb movements during sleep (PLMS) are present in 80% of patients with RLS^11^ and often associated with micro-arousals, that may contribute to sleep fragmentation and repeated increases of blood pressure (BP) and heart rate (HR) throughout the night^12–15^. The association between nocturnal BP elevations and PLMS in patients with RLS could increase the frequency of blunted BP day-to-night dip, nocturnal hypertension and cardiovascular morbidity and mortality^16–21^. However, only two studies have investigated the relationships between RLS and non-dipping BP profile (<10% BP drop during nighttime) by 24-hour ambulatory BP monitoring (ABPM)^22,23^ with contradictory results. Moreover, they did not use polysomnography (PSG) to assess PLMS presence, and RLS symptoms were screened by self-reported questionnaires without confirmation with a face-to-face clinical interview. Finally, a recent study^24^ found that vascular endothelial dysfunction, another predictive marker of CVD morbidity and mortality^25,26^, is more common among patients with RLS than controls.

Therefore, to thoroughly analyze the dipping BP pattern, HR profile and endothelial function in RLS, we compared the 24-hour BP and HR variations, the night-to-day BP ratio, the non-dipping pattern and endothelial function in drug-free adult patients with primary RLS and controls after PSG assessment. Our aim was also to identify the clinical determinants of abnormal BP and HR regulation in RLS.

## Results

### Clinical and PSG characteristics

The mean ages at RLS onset and at RLS diagnosis were 40.6 (±15.8) years and 49.7 (±12.8) years, respectively. Patients reporting moderate, severe and very severe RLS symptoms were 9 (12%), 39 (52%) and 27 (36%) respectively. Age, sex, BMI, smoking history, and rate of hypertension, diabetes and CVD were not significantly different between patients and controls, whereas dyslipidemia was slightly more frequent in the RLS group (p = 0.06) (Table 1). Five of the ten patients (11.9%) and seven of the nine controls (11.8%) with hypertension received an antihypertensive treatment. Among the patients with RLS, 43 (51.2%) were RLS treatment-naive at study entry and 41 (48.8%) medication-free at least for two weeks prior to the evaluation (28 having low-dose dopamine agonists, 9 low-dose dopamine agonists and opioids, and 4 low-dose dopamine agonists, alpha-2-delta ligand and opioids).

**Table 1:** Demographic, clinical and polysomnographic sleep characteristics of patients with restless legs syndrome (RLS) and controls.

	*Controls*		*RLS*		*p*
	*N* = 76		*N* = 84		
	*n*	%	*n*	%	
Sex (female)	47	61.84	53	63.10	0.87
Age, *years*^(1)^	52.17 (±15.30)		55.14 (±12.32)		0.18
Smoker(yes)	12	25.53	14	18.67	0.37
BMI, *kg/m*^2(1)^	24.12 (±3.98)		24.74 (±3.88)		0.32
Hypertension (yes)	9	11.84	10	11.90	0.99
Diabetes mellitus (yes)	2	2.63	4	4.76	0.49
Dyslipidemia (yes)	5	6.58	14	16.67	0.06
CV diseases ^(2)^(yes)	2	2.74	7	8.43	0.15
**Polysomnography**	*N* = 64		*N* = 84		
Total sleep time, *minutes*	362.44 (±65.41)		329.83 (±89.84)		0.02
Sleep efficiency^(1)^, %	75.28 (±12.52)		69.93 (±17.55)		0.04
N1 (%)^(1)^	7.80 (±5.62)		9.48 (±8.49)		0.19
N2 (%)^(1)^	54.06 (±7.44)		51.12 (±10.69)		0.07
N3 (%)^(1)^	21.14 (±7.03)		19.86 (±9.11)		0.35
REM sleep (%)^(1)^	16.98 (±5.19)		19.51 (±7.60)		0.03
Nighttime sleep latency, *minutes*^(1)^	24.11 (±27.77)		18.82 (±23.09)		0.22
WASO, *minutes*^(1)^	87.91 (±47.37)		115.73 (±78.65)		0.02
AHI, */hour*^(1)^	6.64 (±6.51)		7.47 (±6.96)		0.46
PLMS index, */hour*^(1)^	5.59 (±11.28)		51.63 (±56.23)		<0.0001
PLMS index(>15*/hour*)	9	14.06	64	76.19	<0.0001
PLMA index, */hour*^(1)^	1.55 (±3.21)		21.51 (±33.41)		<0.0001
PLMA index(>5*/hour*)	4	6.25	57	67.86	<0.0001
PLMW index, */hour*^(1)^	17.19 (±22.24)		40.05 (±35.29)		<0.0001
Micro-arousal index/hour^(1)^	17.21 (±8.61)		35.32 (±33.78)		0.0002
Mean SaO_2_, %^(1)^	95.03 (±1.44)		95.21 (±1.54)		0.46
Time spent with SaO_2_ < 90%, min^(1)^	0.17 (±0.56)		3.78 (±27.81)		0.17

^(1)^Continuous variables were expressed as mean (±SD).

^(2)^CV, Cardiovascular diseases (myocardial infarcts, arrhythmia, chronic heart failure and stroke).

PSG assessment revealed that total sleep time and sleep efficiency were decreased in the RLS group compared with the controls, whereas REM sleep percentage, wake time after sleep onset (WASO) duration, PLMS, PLMA, PLMW and micro-arousal indexes were increased in RLS patients. In particular, 76.2% of patients had a PLMS index > 15/h, and only 14.1% of controls (p < 0.0001) (Table 1). Seven (10.94%) controls and 13 (15.48%) RLS patients had moderate OSAS (none with severe OSAS), without between-group differences.

### BP monitoring and endothelial function

The mean 24-hour, daytime and nighttime SBP, DBP, HR values were similar in patients and controls, even after adjustment for dyslipidemia (Table 2). Endothelial function did not differ between patients and controls (Table 2). Conversely, SBP and MBP non-dipping profiles were more frequent among patients with RLS than controls (66.7% vs 48.7%, and 57.1% vs 40.9%, respectively, p < 0.05 for both comparisons), especially when non-dipping was defined as a < 15% fall of nocturnal relative to diurnal BP (Table 3). A trend for blunted day-to-night systolic and diastolic dips was found in patients especially in unadjusted model (Table 3).

**Table 2:** Twenty-four hour ambulatory blood pressure monitoring and reactive hyperemia with finger plethysmography in patients with restless legs syndrome and controls.

	*Controls*	*RLS*	*Model 1*	*P*	*Model 2*	*P*
	*Mean* (±SD)	*Mean* (±SD)	*OR* *[95% CI]*		*OR* *[95% CI]*	
**Ambulatory BP**	N = 76	N = 84				
24-hour SBP, *mmHg*	119.33 (±11.61)	118.80 (±13.03)	1.00 [0.97;1.02]	0.79	1.00 [0.97;1.02]	0.73
24-hour DBP, *mmHg*	75.09 (±7.46)	73.80 (±9.28)	0.98 [0.95;1.02]	0.33	0.98 [0.95;1.02]	0.40
**Daytime measures**						
SBP, *mmHg*	122.46 (±11.84)	121.12 (±12.99)	0.99 [0.97;1.02]	0.50	0.99 [0.97;1.02]	0.48
DBP, *mmHg*	78.29 (±7.80)	76.24 (±9.82)	0.97 [0.94;1.01]	0.15	0.98 [0.94;1.01]	0.21
Mean BP, *mmHg*	98.29 (±9.23)	96.31 (±10.08)	0.98 [0.95;1.01]	0.20	0.98 [0.95;1.01]	0.24
Heart rate, */min*	74.39 (±8.46)	74.08 (±9.18)	1.00 [0.96;1.03]	0.82	1.00 [0.96;1.03]	0.93
**Nighttime measures**						
SBP, *mmHg*	111.41 (±13.00)	112.39 (±14.76)	1.01 [0.98;1.03]	0.65	1.00 [0.98;1.03]	0.77
DBP, *mmHg*	67.37 (±8.42)	67.31 (±9.40)	1.00 [0.96;1.03]	0.97	1.00 [0.96;1.03]	0.95
Mean BP, *mmHg*	87.26 (±10.01)	87.58 (±11.31)	1.00 [0.97;1.03]	0.85	1.00 [0.97;1.03]	0.93
Heart rate, */min*	65.16 (±9.62)	62.85 (±8.88)	0.97 [0.94;1.01]	0.12	0.98 [0.94;1.01]	0.21
**Pulse amplitude measures**	N = 69	N = 61				
RH-PAT 90–120 s	2.26 (±0.66)	2.18 (±0.61)	0.81 [0.46;1.40]	0.44	0.86 [0.49;1.51]	0.60
RH-PAT index	0.78 (±0.29)	0.75 (±0.26)	0.66 [0.19;2.35]	0.52	0.76 [0.21;2.78]	0.68

Model 1: Crude associations.

Model 2: Adjustment for dyslipidemia.

BP, blood pressure; DBP, diastolic blood pressure; RH-PAT, reactive hyperemia with finger plethysmography; RLS, primary restless legs syndrome; SBP, systolic blood pressure.

**Table 3:** Blood pressure (BP) dipping profile in patients with restless legs syndrome (RLS) and controls.

	*Controls*		*RLS*		*Model 1*		*Model 2*	
	*N* = 76		*N* = 84					
*Variable*	*n*	%	*n*	%	*OR [95% CI]*	*p*	*OR [95% CI]*	*p*
Systolic dip,%^(1)^	9.03 (±5.93)		7.18 (±7.05)		1.56 [0.95;2.55]^(2)^	0.08	1.49 [0.91–2.46]^(2)^	0.12
**Systolic dip**								
>10%	39	51.32	28	33.33	1	0.02	1	0.04
≤10%	37	48.68	56	66.67	2.11 [1.11;3.99]		1.95 [1.02;3.74]	
**Systolic dip**								
>15%	14	18.42	5	5.95	1	0.02	1	0.03
≤15%	62	81.58	79	94.05	3.57 [1.22;10.4]		3.39 [1.15;9.99]	
Diastolic dip,%^(1)^	13.81 (±8.56)		11.52 (±7.38)		1.44 [0.96;2.16]^(2)^	0.07	1.37 [0.91–2.06]^(2)^	0.13
**Diastolic dip**								
>10%	53	69.74	48	57.14	1	0.10	1	0.15
≤10%	23	30.26	36	42.86	1.73 [0.90;3.32]		1.63 [0.84;3.15]	
**Diastolic dip**								
>15%	36	47.37	28	33.33	1	0.07	1	0.14
≤15%	40	52.63	56	66.67	1.80 [0.95;3.41]		1.64 [0.85;3.14]	
Mean BP dip, %^(1)^	11.17 (±6.75)		9.03 (±7.00)		1.58 [0.99;2.52]^(2)^	0.05	1.49 [0.93–2.39]^(2)^	0.10
**Mean BP dip**								
>10%	45	59.21	36	42.86	1	0.04	1	0.08
≤10%	31	40.79	48	57.14	1.94 [1.03;3.63]		1.77 [0.93;3.37]	
**Mean BP dip**								
>15%	26	34.21	10	11.90	1	0.001	1	0.003
≤15%	50	65.79	74	88.10	3.85 [1.71;8.67]		3.54 [1.56;8.05]	

^(1)^Continuous variables are expressed as mean (±SD).

^(2)^OR for 10-unit decreased.

Model 1: Crude associations.

Model 2: Adjustment for dyslipidemia.

**Figure 1:**
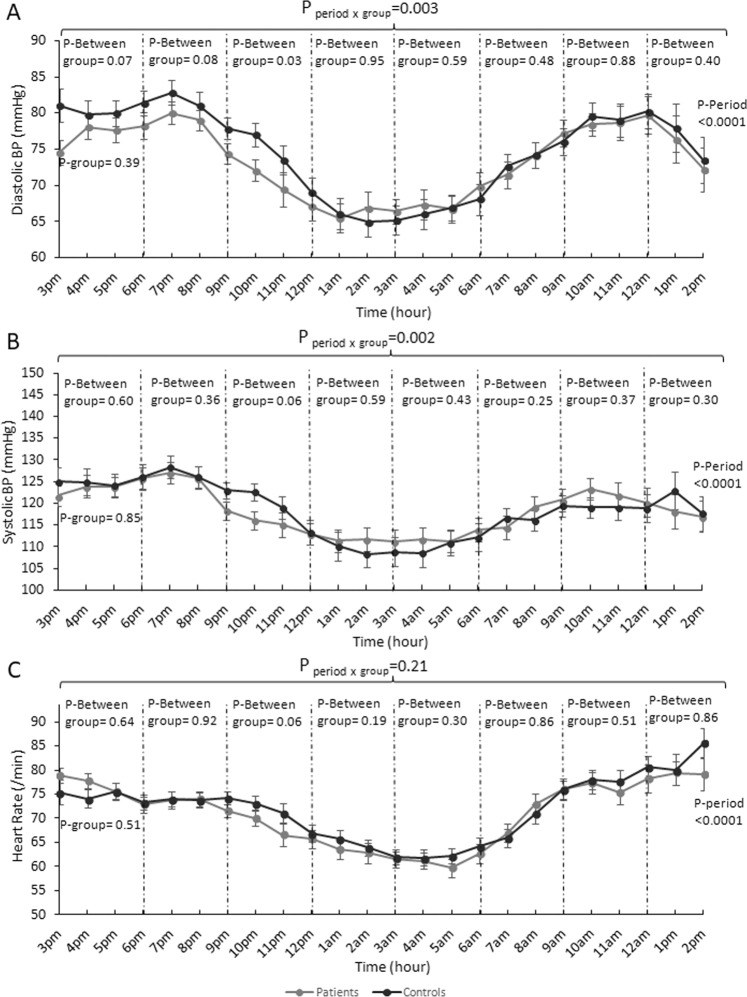
Diastolic and systolic blood pressure and heart rate changes during the 24-hour assessment in patients with restless legs syndrome (RLS) and in healthy controls. Twenty-four hour ambulatory blood pressure monitoring (divided in time periods of 3 hours starting at 3 pm). Mixed models were used to analyze the 24-hour changes in systolic and diastolic BP and heart rate. “Between-group p-values” were calculated for each period. Variation over time within each group was examined over the whole period (“Period p-values”). The difference in change over the 24-hour between the two groups is referred to as “period x group p-values”.

To compare the BP and HR variations in the two groups during the 24-hour ABPM, eight time periods of 3 hours were categorized (Fig. 1). Mixed-effect regression models showed that SBP and DBP trajectories over the 24-hour period differed in the two groups (p < 0.0001 for both variables). Interactions between groups and the eight 3-hour periods were found for SBP (p = 0.002) and DBP (p = 0.003), but not for HR (p = 0.21), indicating differences in the BP trajectories between the two groups. In particular, during the late evening period (9 pm-midnight) patients had lower DBP (p = 0.03) with similar patterns for HR and SBP during this period (p = 0.06) (Fig. 1).

A sensitivity analysis in which only patients with PLMS index > 15/h (n = 64) were compared to controls without PLMS (n = 55) confirmed the higher frequency of systolic and mean BP non-dipping profile in patients with RLS (OR = 2.27, 95%CI [1.11;4.63], p = 0.02; and OR = 2.17, 95%CI [1.08;4.37], p = 0.03, respectively), even after adjustment for dyslipidemia. An additional sensitivity analysis, in which participants with factors potentially associated with a BP non-dipping profile (cardiovascular drug intake, clinical hypertension, CVD, obesity and AHI > 15/h) were excluded (21 RLS patients and 17 controls), found no between-group differences for the mean 24-hour, daytime, nighttime, and nocturnal dip for SBP, DBP and MBP, and for endothelial function.

Comparing RLS patients with (n = 21) and without (n = 63) CV risk factors showed significant greater nighttime SBP in the former group (OR = 1.04 [1.00;1.08]; p = 0.0337), without significant between-group differences for daytime BP, systolic and diastolic non-dipper status, nocturnal dip for SBP, DBP, MBP, and endothelial function. Finally, no significant differences were found for systolic and diastolic non-dipper status, nocturnal dip for SBP, DBP, MBP, nighttime and daytime BP, and endothelial function between drug naïve (n = 43) and drug-free RLS patients (n = 41) for at least two weeks prior to the evaluation.

### Effects of PLMS, PLMA and total sleep time on BP profile in the whole population

As PLMS and PLMA indexes and total sleep time differed between RLS and control groups, we further analyzed their relationship with BP profiles in the whole population. All subjects with PLMS index < 15/hour (n = 75) were compared to participants with PLMS index ≥ 15/hour (n = 73); subjects in the latter group were older (p = 0.02), had higher BMI (p = 0.01), more severe RLS symptoms (p < 0.0001) and more CVD (p = 0.04). No between-group differences were observed for 24-hour, daytime, and nighttime SBP and DBP, or dipping profile frequency. In the same way, we compared participants with PLMA index < 3/hour (i.e. median of the whole sample) to those above, and found that subjects with highest PLMA had higher BMI, were more likely RLS patients, and non-dippers for SBP and MBP, even after adjustment for BMI (p < 0.05 for all these comparisons).

Compared with participants with a total sleep duration ≥335.5 minutes (median threshold of the distribution; n = 74), participants with a shorter sleep duration (n = 74) were older (p = 0.02). Conversely, 24-hour, daytime, nighttime SBP and DBP and dipping profile were not different between groups in unadjusted and adjusted models.

### Determinants of the 24-hour hypertension profile in patients with RLS and controls

The 24-hour ABPM diagnosed hypertension in 39 (46.4%) patients with RLS (28.6% based on the 24-hour, 22.6% on the daytime and 41.7% on the nighttime values) and 36 (47.4%) controls (30.3% based on the 24-hour, 25.0% on daytime and 43.4% on nighttime values).

Hypertensive patients with RLS were more likely to be men, with higher AHI and lower mean oxygen saturation (for daytime and nighttime hypertension), older, with later RLS onset and diagnosis, and with higher ferritin levels (for nighttime hypertension) than normotensive patients with RLS. Analysis of the factors associated with the dipping profile in the RLS group (Table 4) indicated that the diastolic non-dipping profile was associated with older age at the time of the study, at RLS onset and at diagnosis, as well as with RLS severity, longer WASO duration, and higher AHI and micro-arousal indexes. The N1 percentage (p = 0.05), and the PLMA index (p = 0.06) also tended to be higher. In patients with RLS, the systolic non-dipping profile was associated with higher N1 percentage and micro-arousal index. A similar tendency was found for association with older age at RLS onset and at diagnosis (p = 0.05) and increased PLMA and PLMS indexes (p = 0.05 and p = 0.08 respectively). No association was found between endothelial function and systolic or diastolic non-dipping profile in RLS patients (Table 4).

**Table 4:** Determinants of systolic and diastolic nocturnal dip in patients with restless legs syndrome (RLS).

	*SBP Dipping* (*10%*)				*P*	*DBP Dipping* (*10%*)				*p*
	*Yes*		*No*			*Yes*		*No*		
	*N* = 28		*N* = 56			*N* = 48		*N* = 36		
	*n*	%	*n*	%		*n*	%	*n*	%	
Age, *years*^(1)^	52.34 (±11.23)		56.54 (±12.70)		0.14	51.71 (±12.09)		59.71 (±11.22)		0.005
Sex (Male)	11	39.29	20	35.71	0.75	21	43.75	10	27.78	0.14
Current smoker (Yes)	5	20.00	9	18.00	0.83	9	20.93	5	15.63	0.56
BMI, *kg/m*^2^	24.83 (±4.32)		24.69 (±3.68)		0.87	24.50 (±3.98)		25.05 (±3.78)		0.51
Ferritinemia	133.36 (±97.90)		190.25 (±133.21)		0.06	174.94 (±136.83)		166.42 (±108.73)		0.76
Diabetes mellitus (Yes)	1	3.57	3	5.36	0.72	0	0.00	4	11.11	NA
Dyslipidemia (Yes)	2	7.14	12	21.43	0.11	5	10.42	9	25.00	0.08
CV diseases^(2)^ (Yes)	3	11.11	4	7.14	0.55	4	8.51	3	8.33	0.98
Age at RLS onset, *y*^(1)^	35.82 (±15.38)		43.05 (±15.65)		0.05	37.35 (±13.93)		45.03 (±17.32)		0.03
Age at RLS diagnosis, *y*^(1)^	45.19 (±12.66)		51.69 (±12.48)		0.05	45.08 (±12.86)		55.07 (±10.58)		0.002
RLS severity	27.32 (±5.89)		28.10 (±6.26)		0.60	26.48 (±5.86)		29.77 (±6.02)		0.02
Total sleep time^(1)^, *min*	354.07 (±73.55)		317.71 (±95.27)		0.09	341.75 (±84.50)		313.94 (±95.38)		0.16
Sleep efficiency^(1)^, %	74.90 (±13.12)		67.44 (±19.01)		0.07	72.75 (±15.78)		66.17 (±19.24)		0.09
N1 (%)^(1)^	6.91 (±3.42)		10.76 (±9.90)		0.03	7.79 (±3.80)		11.73 (±11.93)		0.05
N2 (%)^(1)^	50.75 (±9.99)		51.30 (±11.10)		0.83	51.28 (±10.31)		50.90 (±11.31)		0.87
N3 (%)^(1)^	22.46 (±6.94)		18.57 (±9.82)		0.07	20.59 (±8.76)		18.89 (±9.59)		0.40
REM sleep (%)^(1)^	19.79 (±5.97)		19.37 (±8.34)		0.81	20.29 (±7.27)		18.47 (±8.00)		0.28
REM sleep latency^(1)^, *min*	100.52 (±56.52)		136.64 (±89.70)		0.07	112.36 (±62.31)		141.67 (±101.48)		0.12
Sleep latency, *min*^(1)^	14.79 (±8.39)		20.84 (±27.52)		0.29	19.35 (±23.29)		18.11 (±23.13)		0.81
WASO^(1)^, *min*	94.07 (±57.61)		126.76 (±85.83)		0.08	98.52 (±64.09)		139.34 (±90.83)		0.03
AHI, */hour*	5.45 (±5.39)		8.48 (±7.46)		0.07	6.07 (±5.76)		9.33 (±8.00)		0.04
PLMS index, */hour*	35.72 (±30.67)		59.58 (±64.16)		0.08	42.86 (±38.66)		63.32 (±72.42)		0.11
PLMS index (>15/h)	20	71.43	44	78.57	0.47	37	77.08	27	75.00	0.82
PLMA index, */hour*	10.12 (±12.06)		27.20 (±38.92)		0.05	15.14 (±18.00)		30.00 (±45.63)		0.06
PLMW index, */hour*	33.24 (±26.61)		43.33 (±38.58)		0.23	39.55 (±31.98)		40.70 (±39.66)		0.88
Microarousal index/hour^(1)^	21.28 (±13.62)		42.34 (±38.48)		0.01	28.02 (±20.56)		45.06 (±44.36)		0.04
Mean SaO_2_, %^(1)^	95.14 (±1.63)		95.25 (±1.50)		0.76	95.40 (±1.47)		94.97 (±1.61)		0.21
**Time spent with**										
SaO_2_ < 90%, min^(1)^	0.32 (±1.15)		5.55 (±34.13)		0.34	0.24 (±0.90)		8.64 (±42.69)		0.11
RH-PAT 90–120 s	2.12 (±0.50)		2.21 (±0.66)		0.73	2.18 (±0.63)		2.18 (±0.59)		0.96
RH-PAT index	0.72 (±0.23)		0.76 (±0.27)		0.73	0.74 (±0.26)		0.75 (±0.26)		0.93

^(1)^Continuous variables were expressed as means (±SD).

^(2)^CV,cardiovascular diseases (myocardial infarcts, arrhythmia, chronic heart failure and stroke).

Hypertensive control subjects were older and showed higher BMI and ferritin levels, lower sleep efficiency, shorter total sleep time (for daytime and 24-hour hypertension), longer WASO duration (for nighttime and daytime hypertension) and were more frequently men (nighttime hypertension only). No risk factor was identified between SBP and DBP dipping and non-dipping profile in the control group.

## Discussion

The ABPM is the most sensitive tool to diagnose hypertension and allows to compare the 24-hour BP and HR profiles in RLS patients and controls. We found that RLS patients were more frequently SBP and MBP non-dippers with a different 24-hour BP trajectory. Conversely, the mean 24-hour, daytime, and nighttime BP and HR, the day-to-night BP dip and endothelial function were similar in the two groups.

The analysis of BP trajectories over the 24-hour period indicated that DBP was significantly lower in patients with RLS than in controls, between 9 pm and midnight, with a similar tendency for SBP. This observation was unexpected because evening is typically the symptomatic period in RLS patients, in which patients experience uncomfortable sensations and need to stretch and move the legs and sometime even walk. On the other hand, this result may suggest a circadian autonomic BP dysfunction, as already described for sensorimotor symptoms and spinal excitability in RLS^27,28^.

Hypertension was diagnosed in only 11.9% of patients with RLS but also in 11.9% of the controls based on office measurements, while it was detected in 46.4% of patients and 47.4% of controls on the basis of the 24-hour ABPM, with a two-fold increase of nighttime compared with daytime hypertension (41.7% vs. 22.6% for RLS patients and 43.4% vs 25% for controls).

Recent large cross-sectional and longitudinal studies found independent associations between RLS, hypertension and CVD^29–32^. Population-based and longitudinal follow-ups of clinical case series on normotensive and hypertensive patients^33,34^ indicate that the loss of the nocturnal BP dip is one of the most sensitive predictor of CV morbidity and mortality, even better than daytime BP. Here, we found that drug-free patients with RLS have a frequent SBP and MBP non-dipping profile (66.7% and 57.1% respectively). This finding confirmed previous results found in patients evaluated for hypertension who were screened for RLS symptoms using only a self-report questionnaire^22^.

The mechanisms underlying the association between blunted nocturnal BP dip, nocturnal hypertension and CVD morbidity in RLS remain unclear; however, PLMS and sleep fragmentation could play a role^9,32^. PLMS are often concomitant with micro-arousals (PLMA), thus contributing to sleep fragmentation and to repeated BP and HR increases throughout the night^12–15^, A recent PSG study with beat-to-beat estimate of BP monitoring using noninvasive pulse transit time showed higher nocturnal SBP, without differences for DBP and HR in patients with RLS than in patients with insomnia^35^ This finding was explained by the increased PLMS and PLMA indexes in patients with RLS, without any association with other sleep disturbances that are reported also by patients with insomnia. The present study confirmed the high PLMS, PLMA and PLMW indexes as well as the increased values for markers of sleep fragmentation (WASO, N1%, micro-arousal index) in RLS. Moreover, nighttime hypertension and diastolic and systolic non-dipping profiles were associated with older age, older age at RLS onset and diagnosis, and also with RLS severity (but only for the diastolic non-dipping status). Previous studies also showed association between RLS severity and non-dipping profile in RLS patients screened by self-reported questionnaires^23^, and increased risk of CVD mortality in patients with longer RLS duration^31^. Altogether, these results highlight the need of a correct and early diagnosis of RLS, especially in severely affected and elderly subjects among whom the risk of hypertension and CVD is higher.

Other factors associated with the non-dipping profile were higher WASO, N1%, AHI and micro-arousal indexes in patients with RLS and controls. This confirms the key influence of sleep fragmentation, and PLMS and PLMA on the 24-hour BP deregulation. A recent large prospective community-based study with an 8-year follow-up reported that RLS, RLS-related sleep disturbances, and PLMS are independent risk factors for incident myocardial infarction^32^. However, in our study, comparison of participants (both patients and controls) with a total sleep duration ≥ or <335.5 minutes did not highlight between-group differences for 24-hour, daytime and nighttime SBP and DBP, or dipping profile. Similarly, comparison of participants with PLMS index above and below the 15/hour threshold did not show any between-group difference for 24-hour, daytime and nighttime BP, or dipping status. Conversely, age, BMI and CVD rate were higher in the group with PLMS index > 15/hour. Another study also reported that PLMS are associated with older age, higher BMI, and history of hypertension in patients with ischemic stroke or transient ischemia attack, without any difference for the 24-hour BP parameters between patients with and without PLMS^36^. These negative findings were attributed to a greater use of antihypertensive drugs among patients with PLMS; however, in our study, only five patients (6%) with RLS were treated with antihypertensive drugs. We also compared here participants with PLMA index above and below the 3/hour threshold (i.e. median of the whole sample), and found higher non-dipper profiles for SBP and MBP in those with higher PLMA. These results emphasize the key role of PLMA instead of PLMS in the blunted BP nocturnal fall, potentially in increasing nocturnal BP and HR elevations associated with sleep fragmentation.

The mechanisms by which RLS predispose to 24-hour BP deregulation may also involve other factors, such as sympathovagal imbalance, altered baroreflex sensitivity, insulin resistance, high catecholamine levels, iron deregulation, oxidative stress, chronic inflammation, genetic factors, and endothelial dysfunction^9,37–42^. In our study, endothelial function did not differ between patients with RLS and controls, suggesting normal arterial smooth muscle cell function in RLS^26^. These results may be also explained by the relatively young age range of the subjects and by the fact that the majority had no CVD risk factors. Our results differ from two previous small studies from the same group on patients with RLS without PSG assessment that showed poor endothelial function being not linked to RLS severity and duration^24,43^. They used a different technique to measure peripheral endothelial function (i.e. ultrasound measurements of the brachial artery flow-mediated dilatation) with key differences between assessments, results and relationships with cardiovascular risk factors^44^. We used the digital automated valid PAT (peripheral arterial tonometry) technique that assesses the microvasculature while they used a challenging less standardized technique that assesses the macrovascular vessels. Moreover, the digital microvessel dilatation we measured seems only partly dependent on nitric oxide in contrast to the brachial flow-mediated vasodilatation highly dependent on nitric oxide. Altogether, these two techniques assessing peripheral endothelial function likely measured different aspects of vascular biology and function.

The present study has several strengths: all participants had complete data on the RLS phenotype, hypertension, CVD and related-risk factors, medication intake and BP monitoring. All RLS patients were drug-free for their disorder at time of ABPM and PSG. PSG data were also available for 84.2% of controls, and endothelial function was assessed in 72% of patients and 91% of controls with a reliable method^26^. ABPM was started the day after the PSG. Therefore, although the night-to-night variability of PLMS and total sleep time in RLS is well known^45^, these parameters assessed in the sleep laboratory could not be directly linked to the BP values recorded by 24-hour ambulatory monitoring. Although this study is to our knowledge the largest study assessing drug-free RLS patients with PSG, ABPM and endothelial function, the sample size may be considered as relatively small, and this could have led to a lack of statistical power when assessing the effect of PLMS and total sleep time on the non-dipping profile. However, to show a significant difference on the systolic dipper profile between groups with PLMS above and below 15/h with a power of 0.80 and a type I error α = 0.025, more than 460 subjects (and 2300 for diastolic dippers) per group would be necessary, which is almost impossible for such recruitment of drug-free RLS patients. Similar calculations for total sleep time above and below 335.5 minutes (median threshold of the distribution) would lead to the recruitment of 1500 subjects per group to show significant differences on the frequency of systolic dippers (and 1000 for diastolic dippers). The recruitment of participants with rare comorbidities causing vascular dysfunction, other confounding factors such as sleep apnea, all (except dyslipidemia) being equally distributed between the RLS and control groups may have underestimated the burden of the BP non-dipping profiles in patients. Finally, sympathetic activity markers laboratory data (C-reactive protein, cytokines, catecholamines and oxidative stress) and RLS predisposing genetic factors were not evaluated in this study.

To conclude, our case-control study demonstrates that patients affected by RLS exhibit a 24-hour BP deregulation with a higher frequency of SBP/MBP non-dipping profiles. ABPM should become the gold standard tool for BP measurement in RLS. RLS should be diagnosed as early as possible for optimal management given the disease burden and the potential increased risk of hypertension and CVD. Future studies should assess the effect of RLS therapy on nighttime BP, non-dipping profile, endothelial function and long-term CVD risk.

## Methods

### Participants

All patients with RLS were admitted to the Sleep Unit, Department of Neurology, University Hospital Center of Montpellier between 2014 and 2018. They underwent a standardized clinical assessment to confirm the diagnosis of primary RLS and exclude RLS mimics, according to standard criteria^1^. Data were collected on all major associated conditions such as hypertension, sleep apnea syndrome, CVD, neurological, liver and renal diseases, diabetes and dyslipidemia, using a semi-structured interview. All medications, including psychotropic and cardiotropic drugs, taken during the previous month were recorded as well as their schedule, dosage and age at intake onset. None of the patients took drugs known to worsen (antidepressant, antihistaminic or antipsychotic drugs) or to alleviate (dopaminergic agonists, levodopa, α2δ ligands, clonazepam and opioids) RLS symptoms for at least two weeks or the equivalent to five half-lives of the drug prior to sleep recording. Patients with the following conditions suggestive of comorbid RLS were excluded: iron deficiency (defined as serum ferritin levels <50 ng/ml), iron overload disorders, pregnancy, chronic kidney or liver diseases, inflammatory, psychiatric or neurological diseases (Parkinson’s disease, multiple sclerosis, polyneuropathy, fibromyalgia, dementia, myelitis, spinal cerebellar ataxia, narcolepsy, REM sleep behavior disorder). RLS severity was evaluated according to the International Restless Legs Syndrome Study Group (IRLSSG) rating scale (IRLSSG score < 10: mild, 10–20: moderate, >20 severe, and >30 very severe RLS); IRLSSG below 15 was an exclusion criteria to participate in this study^46^. All patients underwent one-night PSG recording in the sleep laboratory Sleep Disorders Center, Montpellier-France. Patients with untreated severe sleep apnea syndrome (apnea-hypopnea index, AHI ≥ 30/hour) were excluded, whereas three patients effectively treated with continuous positive airway pressure at the time of the study (AHI < 10/h based on PSG) were retained.

Of the 121 patients screened, 37 patients were excluded with regard to inclusion or exclusion criteria, unavailable data, and refuse to participate. Eighty-four drug-free patients with primary RLS (53 women; mean age 55.1 ± 12.3 years) who fulfilled selection criteria were included.

Control subjects were recruited through advertisements to participate in the study. Inclusion criteria were: normal neurological examination, absence of sleep complaints, normal and regular sleep schedule, and absence of present or past personal history of RLS. Exclusion criteria were: history of renal, neurological, liver, iron, or inflammatory disorders, and any psychotropic drugs intake including dopamine agonists, alpha-2-delta ligand and opioids. Of the 99 controls screened, 23 were excluded and 76 included (47 women; mean age 52.2 ± 15.3 years) to be matched with RLS patients on age, sex and BMI.

The institutional review boards of the University of Montpellier-France approved this study (CPP Sud-Méditerranée IV, Reference 12 03 02). The methods were carried out in accordance with the approved guidelines. Each participant signed legal consent forms. Informed consent was obtained from all subjects.

### Outcomes

The primary efficacy outcome was the between-group (primary RLS patients and controls) difference in the percentage of BP non-dippers status (assessed with ABPM). Secondary endpoints included between-group differences in daytime, nighttime, 24-h BP, dipping status and HR expressed as continuous variable, and endothelial function (assessed with RH-PAT). Additional analyses were performed to identify the clinical and polysomnographic determinants of abnormal BP and HR status in RLS.

### Polysomnography

All patients and 64 controls (84.2%) underwent one-night PSG recording in the sleep laboratory. This included electroencephalogram leads (C3/A2, C4/A1, O2/A1), electrooculography, chin electromyography (EMG) and electrocardiogram. Respiration was monitored with a nasal cannula/pressure transducer system, mouth thermistor, chest and abdominal bands, and pulse oximeter. Leg movements were evaluated with surface EMG electrodes placed on the right and left anterior tibialis muscles. Sleep stages (N1, N2, N3 and REM sleep) were scored manually as well as micro-arousals, PLMS, PLMS associated with micro-arousals (PLMA), PLM during wakefulness (PLMW) and respiratory events (AHI) according to standard criteria^47,48^.

### Ambulatory blood pressure monitoring (ABPM)

ABPM allows to measure BP at regular intervals over 24 hours, using a validated automatic oscillometer (i-MAPA®, Eutherapie). ABPM study started the day after the PSG, and participants were instructed to continue their usual daily activities. BP and HR readings were automatically recorded on the non-dominant arm during daytime (from 7:00am to 11:00 pm) and at 30-minute intervals at night (from 11:00 pm to 7:00am). For each subject, at least 6 nighttime and 16 daytime measures were required. All recorded data were systematically analyzed starting from 3 pm to obtain the mean 24-hour, daytime and nighttime systolic (SBP), diastolic (DBP) and mean blood pressure (MBP, defined as [SBP + 2 (DBP)]/3), and mean 24-hour HR. SBP, DBP and MBP were used to assess the nocturnal dipping status. The BP dip was defined as the difference between the mean daytime and mean nighttime BP, expressed as a percentage of the mean daytime. A “BP non-dipping profile” was defined as a BP dip < 10%^21^. Ambulatory hypertension was defined as a mean 24-hour BP value ≥ 130/80 mmHg, mean daytime BP ≥ 135/85 mmHg and mean nighttime BP ≥ 120/70 mmHg^49^.

### Cardiovascular disease risk factors

All participants underwent a physical examination with measurement of the body mass index (BMI) and venous blood sampling after overnight fasting, at 8:00am. Baseline CVD risk factors were defined as: (1) hypertension (SBP ≥ 140 mmHg and DBP ≥ 90 mmHg on office measurement performed three times at 2-min intervals in supine position at 7:30am using a validated semiautomatic oscillometric sphygmomanometer) and/or treatment with antihypertensive drugs; (2) medical history of diabetes mellitus, fasting blood glucose concentration ≥1.26 g/dL, or use of antidiabetic drugs; (3) hyperlipidemia, total serum cholesterol ≥240 mg/dL, triglycerides ≥150 mg/dL, or use of lipid-lowering drugs; (4) personal history of CVD, coronary artery disease (*e*.*g*., angina pectoris, myocardial infarction), chronic heart failure, arrhythmia or stroke; (5) smoking history.

### Endothelial function assessment

Endothelial function was assessed in 61 patients with RLS and in 69 controls using a valid reliable noninvasive and non-operator dependent method^26^. Digital pulse amplitude was measured in both index fingers between 7:00 and 8:00am, after overnight fasting, in supine position, by reactive hyperemia peripheral arterial tonometry (RH-PAT) with an Endo-PAT finger sensor (Itamar Medical Ltd, Caesarea, Israel)^26^. Data were analyzed using an automated algorithm to obtain the average amplitude at 30-second intervals after forearm cuff deflation and up to 4 minutes. The RH-PAT index, a measure of endothelial dysfunction, was calculated as the natural logarithm of the average PAT amplitude at 90–120 seconds (RH-PAT 90–120 s) after cuff deflation, divided by the mean PAT amplitude during the 210 seconds before cuff inflation^26^.

### Statistical analysis

Categorical variables were presented as percentages, and quantitative variables as means and standard deviation (SD). Logistic regression models were used to compare demographic, clinical, PSG, BP monitoring and RH-PAT data between the two groups (patients with RLS and controls). Potential covariates in our study were chosen according to clinically relevant criteria, along with previously identified factors in the literature: demographic characteristics, anthropometric measurement and cardio-vascular risk factors, (Table 1). We did not add some potential covariates being clearly associated with RLS in literature (i.e. PSG parameters: PLMS, PLMA, PLMW, Micro-arousals, sleep efficiency, WASO with large differences between patients and controls) to avoid overadjustment. As one of the limitation of regression analysis is that it can deal with only a limited number of factors when there is a small number of observations (10 or more observations per variable.), we tested before in an univariate analysis if the potential covariates chosen from the literature were associated with the outcome variable (RLS patients vs Controls) at p < 0.10. The p-value cut-off choice of 0.10 was based on the recommendations of several authors to use a significance level higher than 0.05 for variable selection in the univariate analysis because the level of 0.05 can fail to identify variables known to be important^50^. Covariates that were different (p < 0.10) between groups in the univariate analysis were included in the logistic models to analyze the adjusted associations between the BP monitoring and RH-PAT measures and the two groups. The assumption of linearity to the logit it required in logistic regression for independent continuous variables was verified. Mixed-effect regression models were used to examine the SBP, DBP and HR changes during the 24-hour monitoring, by taking into account the repeated measures (eight time periods of 3 hours starting at 3 pm) and the two groups. Participants were considered as random effects. Time periods, groups and their interaction with the time periods were considered as fixed effects. Statistical significance was set at p < 0.05. Statistical analyses were performed with SAS, version 9.4 (SAS Institute, Cary, NC).
